# Profiling toxin genes and antibiotic resistance in *Bacillus cereus* isolated from pre-launch spacecraft

**DOI:** 10.3389/fmicb.2023.1231726

**Published:** 2023-11-15

**Authors:** Barakatullah Mohammadi, Natalia Gorkina, Marco Esteban Pérez-Reyes, Stephanie A. Smith

**Affiliations:** Consumer Food Safety Laboratory, School of Food Science, Washington State University, Pullman, WA, United States

**Keywords:** *Bacillus cereus*, toxin gene, antibiotic resistance, spacecraft, spacecraft assembly facilities

## Abstract

Characterization of the microbiomes of pre-launch spacecraft in spacecraft assembly facilities is an important step in keeping crews healthy during journeys that can last several hundred days in small artificial environments in space. *Bacillus cereus*, a foodborne pathogenic bacterium, has the potential to be a significant source of food contamination in such environments. This bacterium is a spore-forming bacteria that resists different antimicrobial treatments in cleanrooms where spacecraft are assembled. This study evaluated 41 *B. cereus* isolates from four pre-launch spacecraft in spacecraft assembly facilities for their toxin gene profile and antibiotic resistance. Four enterotoxin genes (*hlbC*, *cytK*, *nheA*, and *entFM*) and two emetic toxin genes (*ces* and *CER*) were targeted for chromosomal DNA and plasmid DNA. Results showed 31.7, 7.3, 85, and 41.5% of isolates contained *hblC*, *cytK*, *nheA*, and *entFM*, respectively, in chromosomal or plasmid DNA. Overall, 37 isolates (90.2%) showed at least one enterotoxin gene. The emetic toxin gene, *ces*, was detected in the plasmid DNA of three isolates (7.3%). The antibiotic resistance of isolates was evaluated by the Kirby-Bauer disk diffusion procedure. All the isolates exhibited 100% susceptibility to gentamicin, 97% were susceptible to clindamycin, and 95% to chloramphenicol, imipenem, tetracycline, and vancomycin. The overall susceptibility average is 51%. However, 98% of the isolates were resistant to β-lactam antibiotics, 97.5% were resistant to sulfamethoxazole/trimethoprim, and 80% were resistant to rifampin. This study provides important information on *B. cereus* isolates from spacecraft assembly facilities for use in microbial monitoring programs of spacecraft.

## 1. Introduction

The main objective of manned space missions is to establish a long-term presence in space, in orbiting stations or planetary bases on the moon and/or Mars. Such missions can only be carried out if the astronauts’ safety and well-being are guaranteed (Novikova et al., 2006). Microbial contamination of these stations is a risk. In a study, Novikova et al. (2006) divided sources of microbial contamination of spacecraft facilities into initial and secondary contamination. Initial contamination can occur (1) during the initial manufacturing and assembly of space flight materials; (2) during the transporting of materials to the orbital station; and (3) from the contaminated supplies. Secondary contamination can happen throughout the lifetime of the station, from the crew, and from any other biological organisms such as animals, plants, and microorganisms on board (Novikova et al., 2006). Therefore, characterization of the microbial contamination of pre-launch spacecraft is critical in ensuring the safety of the crew in long space flights.

Spacecrafts are assembled in cleanrooms where different microbiological control programs and cleaning processes are applied to spacecrafts before launch to avoid contaminating the missions (Moissl-Eichinger, 2017). Though the condition in cleanrooms is extremely harsh for microbial life because of low nutrient levels, temperature control, humidity control, as well as dry and clean conditions (Moissl-Eichinger, 2017), a variety of spore-forming and non-spore-forming bacteria have been isolated from spacecrafts and assembly facilities.

Multiple investigations into the microbial load of spacecraft, space stations, and assembly facilities have revealed the prevalence of *Bacillus* spp. in these environments. an early study of Viking spacecraft reported the spore-forming *Bacillus* spp. as the second most bacteria isolated, with the Micrococcaceae family being the most predominant (Puleo et al., 1977). Additionally, microbial characterization studies of Odyssey spacecraft and the Kennedy Space Center Spacecraft Assembly and Encapsulation Facility II (SAEF-II) showed *Bacillus* as the most dominant bacteria (La Duc et al., 2003), with Smith et al. (2017) further confirming the prominence of *Bacillus* spp. by reporting that 68.3% of isolates from surface of Mars Science Laboratory belong to *Bacillus* spp. (Smith et al., 2017). However, in case of the Mir spacecraft, *Bacillus* was identified as the third most prevalent bacteria, accounting for 27.5% of the surface, 34.0% of the air, and 12.5% of the condensate environment. *Staphylococcus* and *Corynebacterium* were the first and second most prevalent bacteria, respectively (Novikova, 2004).

These microbial findings raise concerns about the transfer of pathogenic bacteria from contaminated pre-launch spacecraft to space stations, potentially posing risks to crew health and technical equipment. A analysis of International Space Station (ISS) revealed *Bacillus* spp. with 31.7% of the total was the second most frequently found bacterium in potable water, air, and surfaces (Novikova et al., 2006). In another microbial investigation of the ISS, 11 *Bacillus* strains were isolated, where genome-sequencing of these strains showed that they are not toxin-producing (Venkateswaran et al., 2017). *Bacillus cereus*, a pathogenic spore-forming facultatively anerobic bacterium, has been consistently isolated from spacecrafts, assembly facilities, and space stations. Six *Bacillus* strains belonging to *B. anthracis*, *B. cereus*, and *B. thuringiensis* have been isolated from the ISS (Van Tongeren et al., 2017). One strain was isolated from the Japanese Experiment Module, a self-contained living and working area within space stations; three from U.S. Harmony Node 2, a specialized module interconnecting various modules; and two from the Russian Segment, which serves specific functions and houses modules and nodes, specifically from the Zvezda Module (Van Tongeren et al., 2017).

Madrigal et al. (2022) conducted a study *in silico* to characterize the antimicrobial resistance of ISS surface microbiome. The research incorporated existing datasets, a characteristic often associated with *in silico* investigations. Specifically, the study drew upon shotgun metagenomics data originating from the International Space Station (ISS), collected during the MT-1 project. This dataset encompassed metagenomics-assembled genomes from various locations across three different flights spanning a 12-month period. By leveraging this valuable real-word data, we were able to derive the conclusion that in space, microgravity, radiation, and confinement could exacerbate antibiotic resistance and thus make it a significant concern for astronaut health (Madrigal et al., 2022). One response to spaceflight stress is an increase in antibiotic resistance of bacteria that develops from genetic alteration (Morrison et al., 2017). Moatti et al. (1986) described that during ASTP (Apollo-Syouz-Test project), Taylor and Zaloguev found that the bacteria collected from astronauts during the spaceflight are more resistant to antibiotics than are the bacteria isolated from the same people in environments pre-and post- flight. Under space flight conditions, bacteria may also mutate and become more virulent and antibiotic resistant. Harmless bacteria may become potential pathogens (Schiwon et al., 2013).

Studies have analyzed the antibiotic resistance of bacteria related to space flights (Schiwon et al., 2013; Morrison et al., 2017; Singh et al., 2018). In an analysis of the phenotypic, genomic, transcriptomic, and proteomic changes of *B. cereus* after a short-term space flight, Su et al. (2014) found that the *B. cereus* flight isolates had higher antibiotic resistance than did the ground control strain. Astronauts living in space stations suffer from weakened immune systems, possibly due to easier exposure to pathogenic bacteria (Gueguinou et al., 2009 as cited in Su et al., 2014). After prolonged exposure to a space environment, *B. cereus* may pose a direct threat to astronaut health (Su et al., 2014).

To investigate the pathogenicity of *B. cereus* and the threat it poses to space mission flights, we evaluated the toxin gene profile and antibiotic resistance of 41 *B. cereus* isolates that were collected as early as the 1970s from four different spacecraft assembly facilities in the United States. Our objective for evaluating *B. cereus* isolates with a broad age spectrum was to confirm potential differences in both pathogenicity and antibiotic resistance profiles among isolates of varying ages. Moreover, due to data availability constraints, including older isolates enriched our dataset and ensured a sufficient sample size for comprehensive statistical analyses and sound conclusions, especially when recent isolates were access-restricted.

## 2. Materials and methods

### 2.1. Sample collection

In this study, 41 *B. cereus* cultures were used from four different spacecraft assembly facilities located in the U.S. Samples were collected based on NASA Technical Standard procedure [Office of Safety and Mission Assurance (OSMA), 2022]. The 16S rRNA gene was amplified using 8F and 1525R bacterial primers, and PCR was performed to identify the isolates in the University of Idaho (Smith et al., 2017). After 41 isolates were identified as *B. cereus*, a glycerol stock solution of isolates was made and transferred to the Washington State University and stored at −80°C. The samples included two from Kennedy Space Center (KSC), nine from Jet Propulsion Laboratory (Curiosity Rover) identified by numbers like 123.1.2, 27 from different parts of Viking Spacecraft OA (Orbiter A), LA (Lander A), LB (Lander B), and SA (Shroud A), and three from Lockheed Martin Space (Insight Lander) referred to IN. The microbial sample collection occurred during the assembly and pre-launch of the spacecraft while in cleanrooms as previously described by Smith et al. (2017).

### 2.2. Isolation and enumeration of *Bacillus cereus*

Tryptic soy agar (TSA) plates were streaked for isolation from a glycerol stock solution of each isolate. The plates were incubated at 37°C for 24–48 h. A single colony was inoculated into a test tube with 10 mL tryptic soy broth (TSB). The tubes were incubated at 32°C for 16–24 h in a shaking incubator.

### 2.3. Extraction and purification of *Bacillus cereus* DNA

Cells from grown cultures were collected by centrifugation at 4,000 g for 7 min. Chromosomal DNA was extracted and purified with the Wizard SV Genomic DNA Purification System Kit (Promega Corporation, Madison, WI) in accordance with the manufacturer’s instructions. Plasmid DNA was extracted and purified with the Wizard Plus SV Minipreps DNA Purification System Kit (Promega Corporation, Madison, WI) in accordance with the manufacturer’s instructions. Both chromosomal and plasmid DNA were stored at −80°C.

### 2.4. Toxic gene identification

PCR was performed to amplify the DNA for the detection of toxic genes in *B. cereus*. Forward and reverse primers were used for the detection of emetic (*ces* and *CER*) and diarrheal (*hblC*, *cytK*, *nheA*, and *entFM*) genes. Table 1 provides information on the primer sequences, product size, and design sources. PCR reagents were added to each tube in the following volumes: 1 μL of chromosomal or plasmid DNA, 25 μL of Midas Mix 2X Master Mix (Monserate Biotechnology Group, San Diego, CA), 2.5 μL of 12.5 μmol forward primer, and 2.5 μL 12.5 μmol reverse primer. Nuclease-free distilled water was added to bring the final volume up to 50 μL after the addition of chromosomal DNA. PCR conditions were as follows: an initial denaturation step at 94°C for 5 min was followed by 32 cycles of 95°C for 1 min, 51.4°C for 1.5 min, and 72°C for 1.5 min. Completion of the 32 cycles was followed by a final elongation step at 72°C for 5 min. PCR amplified fragments were purified with Exonuclease I (10 U), and Antarctic Phosphatase, (2 U), per 5 μL of PCR product. PCR products were run on a 1% agarose gel to identify amplification of products. PCR reactions with amplicons were then treated prior to sequencing. Briefly, the reaction was heated at 37°C for 15 min followed by heat inactivation of the enzymes at 80°C for 15 min. PCR products underwent Sanger sequencing using the primers described in Table 1 (Psomagen, Rockville, MD). The sequences were analyzed with BLAST (Altschul et al., 1997) to confirm the amplified products.

**Table 1:** PCR primers used in this study as previously described by Kim et al. (2012).

Target Gene	Sequence	Product length	Reference strain accession number
*cytK*	TGCTAGTAGTGCTGTAACTC CGTTGTTTCCAACCCAGT	881	DQ019311
*nheA*	GGAGGGGCAAACAGAAGTGAA CGAAGAGCTGCTTCTCTCGT	750	DQ019312
*ces*	TTCCGCTCTCAATAAATGGG TCACAGCACATTCCAAATGC	634	AY691650
*CER*	GCGTACCAAATCACCCGTTC TGCAGGTGGCACACTTGTTA	546	AY576054
*hblC*	CGCAACGACAAATCAATGAA ATTGCTTCACGAGCTGCTTT	421	AY786407
*entFM*	AGGCCCAGCTACATACAACG CCACTGCAGTCAAAACCAGC	327	AY789084

### 2.5. Antibiotic resistance testing

Antibiotic resistance testing was performed using the Kirby-Bauer disk diffusion procedure as published by the American Society for Microbiology (Hudzicki, 2009). In this protocol, our bacterial cultures were evenly spread on the surface of Mueller-Hinton (MH) plates. Antibiotic disks were then placed on the MH plates, and the plates were incubated for 24–48 h at 37°C. After incubation, the size of the zones of inhibition around each disk is measured. The size of the inhibition zones was compared with zone diameter interpretative standards for Staphylococcus species which is published in the Clinical & Laboratory Standards Institute (CLSI) guidelines.

Thirteen antibiotics (manufactured by Hardy Diagnostics, Santa Maria, CA, USA), ampicillin (AM) (10 μg), oxacillin (OX) (1 μg), chloramphenicol (C) (30 μg), ciprofloxacin (CIP) (5 μg), clindamycin (CC) (2 μg), erythromycin (E) (15 μg), gentamicin (GM) (10 μg), imipenem (IPM) (10 μg), penicillin (P) (10 μg), rifampin (RA) (5 μg), sulfamethoxazole/trimethoprim (SXT) (25 μg), tetracycline (TE) (30 μg), and vancomycin (VA) (30 μg) were used in this study.

Four isolates had difficulty growing on Mueller-Hinton (MH) plates. These isolates (288.1.2, LB 243, OA 003, and KSC 208) were taken from the original TSA plates and placed in TSB tubes. They were then plated on TSA plates as lawn plates and antibiotic disks were added as in the Kirby-Bauer disk diffusion procedure. The plates were incubated for 24 to 48 h at 37°C.

## 3. Results

### 3.1. Toxin gene profile

The present study investigated the genes associated with the production of enterotoxins and emetic toxins, including *hblC*, *cytK*, *nheA*, *entFM*, *ces*, and *CER*. The distributions of these genes in chromosomal and plasmid DNA were analyzed, as shown in Table 2. PCR was used to detect the presence of these genes. Of the 41 isolates tested, 83% were positive for these genes in their chromosomal DNA, while only 10% tested positive for the presence of these genes in plasmid DNA. Most of the plasmid DNA samples were not positive for the genes associated with enterotoxin production. Of the plasmid DNA, four (10%) isolates contained *hblC*, *nheA*, and *entFM*, while only one isolate (2%) contained the *cytK* gene. The most detected gene in chromosomal DNA was *nheA*, with 33/ 41 isolates (80%) testing positive for this gene. In contrast, the least common gene was *cytK*, with only two isolates (5%) having this gene. The *entFM* and *hblC* genes were present in 13 (32%) and 10 (24%) isolates, respectively, representing intermediate levels of prevalence. Some isolates contained multiple genes, with 21 (51%) having only one gene present, 16 (39%) having two genes, and four (10%) having three genes. Overall, most of the isolates tested positive for at least one gene for enterotoxin.

**Table 2:** Toxin gene profile based on PCR results.

Isolates name	Chromosomal DNA						Plasmid DNA					
	Enterotoxin gene				Emetic gene		Enterotoxin gene				Emetic gene	
	*hlbC*	*cytK*	*nheA*	*entFM*	*cer*	*CER*	*hlbC*	*cytK*	*nheA*	*entFM*	*ces*	*CER*
LA 003	−	−	+	+	−	−	−	−	−	−	−	−
LA 092	+	−	+	+	−	−	−	−	−	−	−	−
LA 099	+	−	+	+	−	−	+	+	+	+	−	−
LA 187	+	+	+	−	−	−	−	−	−	−	−	−
LA 257	−	−	−	−	−	−	−	−	−	−	−	−
LA 270	−	−	+	+	−	−	−	−	−	−	−	−
LA 393	−	−	−	+	−	−	−	−	−	−	−	−
LA 395	+	−	+	+	−	−	−	−	−	−	−	−
LA 400	−	−	+	+	−	−	−	−	−	−	−	−
56.1	−	−	+	+	−	−	−	−	−	−	−	−
59.1.1	−	−	−	+	−	−	−	−	−	−	−	−
103.2.1	−	−	+	+	−	−	−	−	−	−	−	−
103.2.2	−	+	+	+	−	−	+	−	+	+	+	−
104.1.1	−	−	+	−	−	−	+	−	+	+	+	−
105.1	−	−	+	−	−	−	−	−	−	−	−	−
227.1.1	−	−	+	−	−	−	−	−	−	−	−	−
227.1.2	−	−	−	−	−	−	+	−	+	+	+	−
288.1.2	−	−	+	−	−	−	−	−	−	−	−	−
LB 073	−	−	+	+	−	−	−	−	−	−	−	−
LB 077	+	−	+	−	−	−	+	−	−	−	−	−
LB 081	+	−	+	−	−	−	+	−	−	−	−	−
LB 082	−	−	+	−	−	−	−	−	−	−	−	−
LB 134	+	−	+	−	−	−	+	−	−	−	−	−
LB 135	−	−	+	−	−	−	−	−	−	−	−	−
LB 136	+	−	+	−	−	−	+	−	−	−	−	−
LB 243	−	−	+	−	−	−	−	−	−	−	−	−
OA 003	−	−	+	−	−	−	−	−	−	−	−	−
OA 018B	−	−	−	−	−	−	−	−	−	−	−	−
OA 086	−	−	+	−	−	−	−	−	−	−	−	−
OA 104	−	−	+	−	−	−	−	−	−	−	−	−
OA 288	−	−	+	−	−	−	−	−	−	−	−	−
SA 005	−	−	−	−	−	−	−	−	−	−	−	−
SA 007	−	−	−	−	−	−	−	−	−	−	−	−
SA 008	+	−	+	−	−	−	+	−	−	−	−	−
SA 010	−	−	+	−	−	−	−	−	−	−	−	−
SA 012	+	−	+	−	−	−	+	−	−	−	−	−
IN 352	−	−	+	−	−	−	−	−	−	−	−	−
IN 383	−	−	+	−	−	−	−	−	−	−	−	−
IN 384	−	−	+	+	−	−	−	−	−	−	−	−
KSC 72	−	−	+	+	−	−	−	−	−	−	−	−
KSC 208	−	−	+	+	−	−	−	−	−	−	−	−
R^a^ (%)	10 (24)	2 (5)	34 (83)	15 (37)	0 (0)	0 (0)	10 (24)	1 (2)	4 (10)	4 (10)	3 (7)	0 (0)

^a^(Number of positives/numbers of total isolates) × 100.

This study also focused on identifying the *ces* and *CER* genes necessary to produce the emetic toxin. Table 2 displays the distribution of these genes in chromosomal and plasmid DNA. Of the 41 isolates, only 10% contained the *ces* gene in plasmid DNA, with no isolates presenting these genes in their chromosomal DNA. None of the samples, whether chromosomal or plasmid DNA, showed the presence of the *CER* gene. Additionally, all the detected *ces* genes were in the plasmid DNA. In summary, the prevalence of emetic toxin genes was minimal in the isolates tested.

### 3.2. Antibiotic resistance

The antibiotic resistance of the isolates was evaluated against 13 antibiotics: ampicillin, oxacillin, chloramphenicol, ciprofloxacin, clindamycin, erythromycin, gentamicin, imipenem, penicillin, rifampin, sulfamethoxazole/trimethoprim, tetracycline, and vancomycin. The findings, presented in Table 3, show that a large majority of the isolates (98%) were resistant to β-lactam antibiotics, specifically ampicillin, penicillin, and oxacillin. Additionally, 97% of the isolates showed resistance to sulfa/trimeth, while 80% exhibited resistance to rifampin.

**Table 3:** *Bacillus cereus* isolates resistance and susceptibility to antibiotics.

Antibiotic type	Antibiotics	No. of isolates (%)		
		Resistant	Intermediate	Susceptible
β-lactam	Ampicillin	40^a^ (98)^b^	0 (0)	1 (2)
	Penicillin	40 (98)	0 (0)	1 (2)
	Oxacillin	40 (98)	0 (0)	1 (2)
Amphenicols	Chloramphenicol	2 (5)	0 (0)	38 (95)
Quinolones	Ciprofloxacin	2 (5)	38 (95)	0 (0)
Lincosamides	Clindamycin	0 (0)	1 (3)	39 (97)
Macrolides	Erythromycin	7 (18)	0 (0)	33 (83)
Aminoglycosides	Gentamicin	0 (0)	0 (0)	40 (100)
Carbapenems	Imipenem	2 (5)	0 (0)	39 (95)
Rifamycins	Rifampin	32 (80)	7 (18)	1 (3)
Folic acid inhibitors	Sulfa/Trimeth	38 (97)	0 (0)	1 (3)
Tetracyclines	Tetracycline	2 (5)	0 (0)	37 (95)
Glycopeptides	Vancomycin	2 (5)	0 (0)	37 (95)
Average		16 (39)	4 (10)	21 (51)

^a^Number of isolates.^b^Percentage of isolates.

However, most of the isolates showed susceptibility to the other antibiotics. The highest susceptibility was observed for gentamicin (100%), while 97 and 95% of the isolates were susceptible to clindamycin and chloramphenicol, respectively. Similarly, imipenem, tetracycline,and vancomycin also exhibited significant susceptibility, with 95% of the isolates being susceptible to these antibiotics. 95% of the isolates fell intermediately between resistance and susceptibility to ciprofloxacin.

## 4. Discussion

### 4.1. Toxin gene profile

Determination of toxin production properties of spacecraft-associated bacteria is essential for keeping astronauts and crew members healthy. Hbl is an enterotoxin with three components, L2, L1, and binding B, which are encoded by Hbl operon, *hblC*, *hblD*, and *hblA*, respectively. Nhe is also a three-component enterotoxin that is encoded by *nheA*, *nheB*, and *nheC* (Mohammadi et al., 2022). In this study we only targeted *hblC* from Hbl operon and *nheA* from Nhe enterotoxin genes. The detection rate of *hblC, cytK, nheA* and *entFM* genes in all isolates were 31.7, 7.3, 85, and 41.5%, respectively. Our findings also point to the possible diarrheal pathogenicity of 90.2% (37 out of 41) of the isolates that had at least one enterotoxin gene. Only four isolates did not show the presence of DNA that encodes for an enterotoxin gene or set of genes. Previous studies have shown that *B. cereus* isolates from a variety of food sources had a high frequency of the *nheA, hblC, cytK*, and *entFM* gene complexes (Ankolekar et al., 2009; Park et al., 2009; Chon et al., 2012; Owusu-Kwarteng et al., 2017; Navaneethan and Effarizah, 2021).

Ankolekar et al. (2009) revealed that 84.3% of *B. cereus* isolates from U.S rice are positive for *nhe* and 61.4% for *hbl*. Similarly, 63% of *B. cereus* isolated from farm and dairy products possessed at least one *hbl* gene, and 60% of these isolates were positive for all three *nhe* genes (Owusu-Kwarteng et al., 2017). Owusu-Kwarteng et al. (2017) also found that the detection rate of *cytK* and *entFM* was 75 and 67%, respectively, in these isolates. Another study conducted on 35 *B. cereus* strains isolated from 100 samples of commercial Sunsik (a ready-to-eat Korean food) shows similar detection rates of *nhe* (97%), *hbl* (86%), *cytK* (77%), and *entFM* (100%) (Chon et al., 2012). High detection rates of *nheA* (99%), and *hblDC* (84%) were reported in *B. cereus* isolates of cereals, while *cytK* shows a lower (55%) detection rate (Park et al., 2009).

The *ces* gene is responsible for the production of cereulide toxin, known as the emetic toxin. This gene was detected in 7.3% (3 out of 41) of the isolates we studied. These results are consistent with previous studies where the *ces* gene was detected in 9% of *B. cereus* strains in dairy products (Owusu-Kwarteng et al., 2017), 2.9% of isolates from Sunsik (Chon et al., 2012), and 14.7% of isolates from cooked rice (Navaneethan and Effarizah, 2021). In other studies, they were unable to detect *B. cereus* isolates containing this toxin, such as no detection in rice samples in the US during a 2009 study by Ankolekar et al., or any *B. cereus* isolates that were collected in another study from the Antarctic Concordia Station and ISS (Timmery et al., 2011). These results indicate that cereulide toxin production appears to have low prevalence among *B. cereus* isolates.

To fully understand the underlying reasons for the absence of the *CER* gene in all of the samples as well as understand why the prevalence of some genes is higher than others, more genetics experiments are necessary. These would encompass mutation analysis, investigations into horizontal gene transfer, assessments of selective pressures, and further genetic studies to explore the factors and environmental conditions contributing to the absence of the *CER* gene. Although our study, constrained by limitations of time and equipment, did not include these experiments, we believe that our results will serve as a valuable foundation for future research endeavors in this domain.

### 4.2. Antibiotic resistance

Understanding whether *B. cereus* is resistant to antimicrobial drugs is crucial to optimize treatment during outbreaks (Owusu-Kwarteng et al., 2017). Levels of resistance and susceptibility of *B. cereus* isolates to antibiotics are presented in Table 3. Our results show that *B. cereus* isolates are highly resistant to β-lactam antibiotics. This resistance is explained by the β-lactamase production of *B. cereus* (Savic et al., 2016). β-lactam antibiotics work by inhibiting the transpeptidases, enzymes that are involved in biosynthesis of bacterial cell walls, from forming cross-linked structures and weakening the bacterial cell walls. However, β-lactamase inactivates the β-lactam antibiotics by hydrolyzing the β-lactam ring. The hydrolysis prevents the β-lactam from binding to transpeptidases, thereby not being able to affect the cell wall synthesis (Reygaert, 2018). The findings in our study are in agreement with previous studies of antibiotic susceptibility of *B. cereus*. Many studies have found that *B. cereus* isolates from different food sources are resistant to β-lactam antibiotics such as penicillin, ampicillin, and oxacillin (Park et al., 2009; Chon et al., 2012; Owusu-Kwarteng et al., 2017; Shawish and Tarabees, 2017; Min Park et al., 2018; Navaneethan and Effarizah, 2021).

In this study, 97% of the isolates were resistant to sulfamethoxazole/trimethoprim and 80% of the isolates to rifampin. Sulfamethoxazole/trimethoprim acts by inhibiting the bacterial enzymes dihydropteroate synthase and dihydrofolate reductase, which are the key enzymes in bacterial folate synthesis. Blocking the folate synthesis prevents bacterial growth and reproduction by inhibiting the nucleic acids formation (Kemnic and Coleman, 2022). However, the resistance mechanism to sulfamethoxazole/trimethoprim is mainly caused by an additional production of dihydrofolate (plasmid-encoded) reductase, and overproduction of plasmid-encoded dihydropteroate synthetase with a reduced affinity for sulfamethoxazole/trimethoprim (Dever and Dermody, 1991).

Additionally, rifampin works by inhibiting bacterial RNA polymerase, which is responsible for transcribing DNA into RNA during gene expression. Specifically, rifampin prevents bacterial growth and reproduction by binding to the beta subunit of bacterial RNA polymerase and blocking its activity, thereby preventing the transcription of bacterial genes (Kohanski et al., 2010). Mutations in bacterial genes encoded for the beta subunit of RNA polymerase form the resistance mechanism against rifampin. These mutations can alter the binding site of rifampin and reduce its affinity for RNA polymerase, thereby reducing its effectiveness. Additionally, bacteria can acquire resistance genes through horizontal gene transfer, which encodes enzymes that modify or degrade rifampin (Peterson and Kaur, 2018).

On the other hand, 100% of isolates showed susceptibility to gentamicin. Gentamicin binds to the 30S subunit of the ribosome, which is responsible for decoding mRNA and assembling amino acids into proteins. By binding to this subunit, gentamicin can cause errors in protein synthesis by producing non-functional or toxic proteins that can ultimately lead to bacterial death (Kohanski et al., 2010). Overall, in this study, the isolates were susceptible to the rest of the tested antibiotics. The susceptibility mechanism of *B. cereus* against antibiotics varies depending on different antibiotics. In general, antibiotics work by disrupting crucial cellular mechanisms in bacteria, such as the synthesis of cell walls, proteins, or DNA replication. This interference inhibits the growth and proliferation of bacteria, eventually resulting in their death (Kohanski et al., 2010).

In this study, the susceptibility average of the isolates (51%) is higher than the overall resistance average (39%). These averages help in providing effective treatment of illnesses, clinical decision-making, and developing public health policies.

## 5. Conclusion

The results of this study show that the majority of *B. cereus* isolates in spacecraft assembly facilities are enterotoxin-producing genes in that they have at least one toxin-producing gene. Emetic toxin-producing strains are fewer than the enterotoxin-producing strains, but they could still significantly contribute to illnesses among astronauts during long-term space flight. Although the susceptibility of these isolates is higher than their resistance against most tested antibiotics, the conditions during space flight can increase the resistance of *B. cereus* isolates against antibiotics (Madrigal et al., 2022). These changes during flight would present challenges in selecting the best antibiotics to treat *B. cereus*-induced illnesses. This study can help the microbial monitoring programs of assembly stations to develop effective interventions to avoid contamination and ensure the crews remain healthy during flights. Further studies could be done on characterization and antibiotic susceptibility of *B. cereus* isolates during and after flights to determine changes in antibiotic resistance and pathogenicity after exposure to space. Additionally, microbial monitoring programs of *B. cereus* isolates at the assembly facilities could help with evaluating risks to human health during pace travel.

## Data availability statement

The data used in this manuscript is stored in the laboratory at the School of Food Science and will be made available in compliance with WSU’s policies upon request to the corresponding author.

## Author contributions

BM, NG, and SS contributed to the conception and design of the study. BM and NG performed the experiments. BM wrote the first draft of the manuscript. NG wrote the method and material section of the manuscript. MP-R and SS revised and reviewed the manuscript. All authors contributed to the article and approved the submitted version.

## References

[ref1] Altschul S. F. Madden T. L. Schäffer A. A. Zhang J. Zhang Z. Miller W.. (1997). Gapped BLAST and PSI-BLAST: a new generation of protein database search programs. Nucleic Acids Res. 25, 3389–3402. doi: 10.1093/nar/25.17.3389, PMID: 9254694 PMC146917

[ref2] Ankolekar C. Rahmati T. Labbé R. G. (2009). Detection of toxigenic *Bacillus cereus* and *Bacillus thuringiensis* spores in U.S. rice. Int. J. Food Microbiol. 128, 460–466. doi: 10.1016/j.ijfoodmicro.2008.10.006, PMID: 19027973

[ref3] Chon J. W. Kim J. H. Lee S. J. Hyeon J. Y. Seo K. H. (2012). Toxin profile, antibiotic resistance, and phenotypic and molecular characterization of *Bacillus cereus* in Sunsik. Food Microbiol. 32, 217–222. doi: 10.1016/j.fm.2012.06.003 22850397

[ref4] Dever L. A. Dermody T. S. (1991). Mechanisms of bacterial resistance to antibiotics. Arch. Intern. Med. 151, 886–895. doi: 10.1001/archinte.1991.00400050040010 2025137

[ref5] Hudzicki J. (2009). Kirby-Bauer disk diffusion susceptibility test protocol. Am. Soc. Microbiol. 15, 1–23.

[ref6] Kemnic T. R. Coleman M. (2022). Trimethoprim sulfamethoxazole. StatPearls 25, 375–380. doi: 10.1542/pir.25.11.375 30020604

[ref7] Kim J. M. Forghani F. Kim J. B. Park Y. B. Park M. S. Wang J.. (2012). Improved multiplex PCR assay for simultaneous detection of *Bacillus cereus* emetic and enterotoxic strains. Food Sci. Biotechnol. 21, 1439–1444. doi: 10.1007/s10068-012-0189-8

[ref8] Kohanski M. A. Dwyer D. J. Collins J. J. (2010). How antibiotics kill bacteria: from targets to networks. Nat. Rev. Microbiol. 8, 423–435. doi: 10.1038/nrmicro2333, PMID: 20440275 PMC2896384

[ref9] La Duc M. T. Nicholson W. Kern R. Venkateswaran K. (2003). Microbial characterization of the Mars odyssey spacecraft and its encapsulation facility. Environ. Microbiol. 5, 977–985. doi: 10.1046/j.1462-2920.2003.00496.x, PMID: 14510851

[ref10] Madrigal P. Singh N. K. Wood J. M. Gaudioso E. Hernández-del-Olmo F. Mason C. E.. (2022). Machine learning algorithm to characterize antimicrobial resistance associated with the international Space Station surface microbiome. BioRxiv 10:134. doi: 10.1186/s40168-022-01332-w, PMID: 35999570 PMC9400218

[ref11] Min Park K. Jeong M. Park K. J. Koo M. (2018). Prevalence, enterotoxin genes, and antibiotic resistance of *Bacillus cereus* isolated from raw vegetables in Korea. J. Food Prot. 81, 1590–1597. doi: 10.4315/0362-028X.JFP-18-205, PMID: 30169119

[ref12] Moatti N. Lapchine L. Gasset G. Richoilley G. Templier J. Tixador R. (1986). Preliminary results of the “Antibio” experiment. Naturwissenschaften 73, 413–414. doi: 10.1007/BF00367282 3531874

[ref13] Mohammadi B. Gorkina N. Smith S. A. (2022). “Pathogenicity, toxin production, control and detection of *Bacillus cereus*” in Foodborne pathogens – recent advances in control and detection. IntechOpen 1–21.

[ref14] Moissl-Eichinger C. (2017). Extremophiles in spacecraft assembly cleanrooms. Environ. Extremes, 253–281. doi: 10.1007/978-3-319-48327-6

[ref15] Morrison M. D. Fajardo-cavazos P. Nicholson W. L. (2017). Cultivation in space flight produces minimal alterations in the susceptibility of *Bacillus subtilis* cells to 72 different antibiotics and growth-inhibiting compounds. Appl. Environ. Microbiol. 83, e01584–e01517. doi: 10.1128/AEM.01584-17, PMID: 28821547 PMC5648920

[ref16] Navaneethan Y. Effarizah M. E. (2021). Prevalence, toxigenic profiles, multidrug resistance, and biofilm formation of *Bacillus cereus* isolated from ready-to eat cooked rice in Penang, Malaysia. Food Control 121:107553. doi: 10.1016/j.foodcont.2020.107553

[ref17] Novikova N. D. (2004). Review of the knowledge of microbial contamination of the Russian manned spacecraft. Microb. Ecol. 47, 127–132. doi: 10.1007/s00248-003-1055-2, PMID: 14994178

[ref18] Novikova N. De Boever P. Poddubko S. Deshevaya E. Polikarpov N. Rakova N.. (2006). Survey of environmental biocontamination on board the international Space Station. Res. Microbiol. 157, 5–12. doi: 10.1016/j.resmic.2005.07.010, PMID: 16364606

[ref19] Office of Safety and Mission Assurance (OSMA) (2022). Implementing planetary protection requirements for space flight. NASA Technical Standard, 1–62.

[ref20] Owusu-Kwarteng J. Wuni A. Akabanda F. Tano-Debrah K. Jespersen L. (2017). Prevalence, virulence factor genes and antibiotic resistance of *Bacillus cereus* sensu lato isolated from dairy farms and traditional dairy products. BMC Microbiol. 17, 65–68. doi: 10.1186/s12866-017-0975-9, PMID: 28288581 PMC5348786

[ref21] Park Y. B. Kim J. B. Shin S. W. Kim J. C. Cho S. H. Lee B. K.. (2009). Prevalence, genetic diversity, and antibiotic susceptibility of *Bacillus cereus* strains isolated from rice and cereals collected in Korea. J. Food Prot. 72, 612–617. doi: 10.4315/0362-028X-72.3.612 19343952

[ref22] Peterson E. Kaur P. (2018). Antibiotic resistance mechanisms in bacteria: relationships between resistance determinants of antibiotic producers, environmental bacteria, and clinical pathogens. Front. Microbiol. 9, 1–21. doi: 10.3389/fmicb.2018.02928, PMID: 30555448 PMC6283892

[ref23] Puleo J. R. Fields N. D. Bergstrom S. L. Oxborrow G. S. Stabekis P. D. Koukol R. C. (1977). Microbiological profiles of the Viking spacecraft. Appl. Environ. Microbiol. 33, 379–384. doi: 10.1128/aem.33.2.379-384.1977, PMID: 848957 PMC170694

[ref24] Reygaert W. C. (2018). An overview of the antimicrobial resistance mechanisms of bacteria. AIMS Microbiol. 4, 482–501. doi: 10.3934/microbiol.2018.3.482, PMID: 31294229 PMC6604941

[ref25] Savic D. Miljkovic-Selimovic B. Lepsanovic Z. Tambur Z. Konstantinovic S. Stankovic N.. (2016). Antimicrobial susceptibility and β-lactamase production in *Bacillus cereus* isolates from stool of patients, food and environment samples. Vojnosanitetski Pregled 73, 904–909. doi: 10.2298/vsp150415134s 29327895

[ref26] Schiwon K. Arends K. Rogowski K. M. Fürch S. Prescha K. Sakinc T.. (2013). Comparison of antibiotic resistance, biofilm formation and conjugative transfer of Staphylococcus and Enterococcus isolates from international Space Station and Antarctic Research Station Concordia. Microb. Ecol. 65, 638–651. doi: 10.1007/s00248-013-0193-4, PMID: 23411852

[ref27] Shawish R. Tarabees R. (2017). Prevalence and antimicrobial resistance of *Bacillus cereus* isolated from beef products in Egypt. Open Vet. J. 7, 337–341. doi: 10.4314/ovj.v7i4.9, PMID: 29296593 PMC5738887

[ref28] Singh N. K. Wood J. M. Karouia F. Venkateswaran K. (2018). Succession and persistence of microbial communities and antimicrobial resistance genes associated with international Space Station environmental surfaces. Microbiome 6, 1–23. doi: 10.1186/s40168-018-0609-y, PMID: 30424821 PMC6234677

[ref29] Smith S. A. Benardini J. N. Anderl D. Ford M. Wear E. Schrader M.. (2017). Identification and characterization of early Mission phase microorganisms residing on the Mars science laboratory and assessment of their potential to survive Mars-like conditions. Astrobiology 17, 253–265. doi: 10.1089/ast.2015.1417, PMID: 28282220 PMC5373329

[ref30] Su L. Zhou L. Liu J. Cen Z. Wu C. Wang T.. (2014). Phenotypic, genomic, transcriptomic and proteomic changes in *Bacillus cereus* after a short-term space flight. Adv. Space Res. 53, 18–29. doi: 10.1016/j.asr.2013.08.001

[ref31] Timmery S. Hu X. Mahillon J. (2011). Characterization of Bacilli isolated from the confined environments of the Antarctic Concordia station and the international Space Station. Astrobiology 11, 323–334. doi: 10.1089/ast.2010.0573, PMID: 21563959

[ref32] Van Tongeren S. P. Torres C. Allen J. Jaing C. Pierson D. Perry J.. (2017). Draft genome sequences from a novel clade of *Bacillus cereus* Sensu Lato strains, isolated from the international Space Station. Genome Announc. 5, e00680–e00617. doi: 10.1128/genomeA.00680-17, PMID: 28798168 PMC5552977

[ref33] Venkateswaran K. Singh N. K. Sielaff A. C. Pope R. K. Bergman N. H. van Tongeren S. P.. (2017). Non-toxin-producing *Bacillus cereus* strains belonging to the *B. anthracis* clade isolated from the international Space Station. Msystems 2, 541–554. doi: 10.1128/9781555819972.ch20 PMC5487513 28680972

